# Frequency-dependent associations of contrast sensitivity and corneal aberrations after SMILE PRO in eyes with high astigmatism

**DOI:** 10.3389/fmed.2026.1867550

**Published:** 2026-07-20

**Authors:** Lan Huong Thi Tran, Thanh Ngoc Tran, Hong-Son Cung, Sam Khanh Tran, Van Trong Pham

**Affiliations:** 1 Department of Ophthalmology and Optometry, Hanoi Medical University, Hanoi, Vietnam; 2 Department of Refraction, Vietnam National Eye Hospital, Hanoi, Vietnam; 3 Department of General Ophthalmology, Cung Hong Son International Eye Hospital, Hanoi, Vietnam; 4 Department of Vitreous and Retina, Vietnam National Eye Hospital, Hanoi, Vietnam

**Keywords:** centration, contrast sensitivity, corneal aberrations, cyclotorsion, high astigmatism, optical zone, SMILE PRO, visual quality

## Abstract

**Background:**

Visual quality after SMILE PRO in eyes with high astigmatism remains incompletely characterized, particularly with respect to contrast sensitivity across spatial frequencies and postoperative anterior corneal aberrations.

**Methods:**

This prospective cohort study included 160 eyes of 102 patients with manifest astigmatism ≥2.00 D who underwent SMILE PRO using the VISUMAX 800. Patients underwent preoperative and postoperative assessment of visual acuity, manifest refraction, mesopic contrast sensitivity at 1.5, 3, 6, 12, and 18 cycles per degree, and anterior corneal wavefront aberrations calculated for a 6-mm pupil. Follow-up was performed at 1 week, 1 month, and 3 months. Generalized estimating equation models were used to account for inter-eye correlation and to evaluate associations between 3-month visual quality outcomes and preoperative refractive characteristics, intraoperative optical zone diameter, decentration category, and cyclotorsion.

**Results:**

At 3 months, refractive outcomes were stable, with 98.8% of eyes achieving uncorrected distance visual acuity of 20/25 or better. Preoperative astigmatism subgroup was not significantly associated with postoperative total higher-order aberrations, coma, spherical aberration, or contrast sensitivity. A larger intraoperatively selected optical zone diameter was consistently associated with lower postoperative anterior corneal aberrations. Preoperative spherical equivalent was associated with total higher-order aberrations and spherical aberration, while absolute cyclotorsion was associated with coma. Exploratory analyses of contrast sensitivity showed frequency-specific nominal associations with preoperative spherical equivalent, cylinder, and decentration category.

**Conclusion:**

At 3 months after SMILE PRO in eyes with high astigmatism, anterior corneal aberration and contrast sensitivity outcomes showed exploratory associations with refractive magnitude and intraoperatively selected optical zone diameter, with additional findings related to cyclotorsion and decentration category. Categorical astigmatism severity was not significantly associated with these outcomes. However, this null finding should be interpreted cautiously because the astigmatism subgroups were imbalanced and relatively few eyes had very high astigmatism. These short-term findings require confirmation in larger comparative studies with longer follow-up.

## Introduction

Visual quality extends beyond visual acuity and encompasses multiple dimensions of optical performance, including contrast sensitivity and wavefront aberrations. These parameters provide a more comprehensive assessment of real-world visual function, particularly in patients undergoing refractive surgery. Optical aberrations have been identified as a key contributor to postoperative visual dissatisfaction, even in the presence of excellent visual acuity (1, 2).

Small incision lenticule extraction (SMILE), as part of Keratorefractive Lenticule Extraction (KLEx) techniques, has gained widespread acceptance due to its biomechanical advantages, reduced incidence of dry eye, and absence of flap-related complications. Since its introduction, SMILE has demonstrated excellent safety, efficacy, and predictability in the correction of myopia and myopic astigmatism (3, 4).

However, astigmatism remains a challenging component in refractive surgery. High astigmatism may affect postoperative visual quality through mechanisms that differ from spherical refractive error. A larger cylindrical component increases the dependence of treatment accuracy on axis alignment, rotational stability, and centration. Even small residual axis errors may reduce astigmatic correction efficiency and alter the postoperative optical profile (5). In addition, higher cylindrical correction may modify lenticule geometry and peripheral transition zones, potentially influencing higher-order aberrations (HOAs) and contrast sensitivity. Therefore, evaluating astigmatism magnitude separately from spherical equivalent may help clarify whether postoperative visual quality is driven by cylindrical severity, spherical refractive magnitude, or intraoperative alignment (6, 7). Various techniques, including corneal markings and head position control, have been used to compensate for astigmatic magnitude and static cyclotorsion; however, the outcomes have been variable, and these approaches have underscored the need for effective automated compensation software (8).

The introduction of the VISUMAX 800 platform has improved surgical precision through enhanced centration guidance and integrated cyclotorsion compensation using CentraLign and OcuLign systems. While early studies have confirmed its refractive accuracy and safety, the factors associated with postoperative visual quality, particularly in eyes with high astigmatism, remain incompletely understood (9, 10).

A separate manuscript from the same prospective cohort, focusing on refractive accuracy, visual acuity, safety, and astigmatic vector outcomes using the Alpins method, is currently under review. Baseline cohort characteristics may overlap between the two manuscripts because they derive from the same study population; however, the primary outcomes, statistical analyses, figures, and interpretation are non-overlapping. The present manuscript addresses a distinct research question by focusing on postoperative visual quality. In this study, visual quality was considered as a composite concept with two distinct components. The optical component was assessed using anterior corneal wavefront aberrations, including total higher-order aberrations, coma, and spherical aberration. Anterior corneal aberrations were selected because SMILE PRO primarily alters the corneal stromal and anterior corneal optical profile. The functional component was assessed using contrast sensitivity across multiple spatial frequencies. Visual acuity was included only as a secondary supportive clinical outcome and was not used as the primary measure of visual quality. In addition, we investigated the associations of preoperative refractive characteristics and intraoperative alignment parameters with postoperative visual quality outcomes.

## Materials and methods

### Study design and participants

This prospective cohort study included patients with high astigmatism (≥ 2.00 D) who underwent SMILE PRO surgery using the VISUMAX 800 at Cung Hong Son International Eye Hospital between December 1st, 2024, and December 31st, 2025. The study followed the tenets of the Declaration of Helsinki and was approved by the Institutional Review Board of Hanoi Medical University (IRB ID: HMUIRB1649). Written informed consent was obtained from all participants prior to enrollment.

All surgical procedures were performed by a single surgeon and all preoperative and postoperative parameter measurements were taken by an experienced optometrist with a standardized protocol to ensure consistency.

Inclusion criteria were patients aged from 18 to 40 years old with myopia up to −10.00 diopters (D) with manifest astigmatism ranging from 2.00 D to 5.00 D, stable refraction defined as a change of no more than 0.50 D within the preceding 6 months, and discontinuation of soft contact lenses for at least 1 week and rigid gas-permeable lenses for at least 4 weeks prior to surgery. Preoperative corrected distance visual acuity (CDVA) was required to be 20/25 or better. The estimated residual stromal thickness (RST) had to be ≥ 250 μm.

Exclusion criteria included previous ocular surgery; irregular corneal tomography; keratoconus or keratoconus suspect; cataract; glaucoma; active vitreoretinal disease; active keratitis; or severe dry eye disease (tear breakup time < 5 s according to JDES/ADES criteria). Additional exclusions included monocular status, systemic diseases that could affect wound healing or refractive stability (such as diabetes mellitus or autoimmune disorders), pregnancy, lactation, and the use of hormonal therapies.

### Preoperative and follow-up examinations

All patients underwent a comprehensive ophthalmic examination, including uncorrected and corrected distance visual acuity (UDVA and CDVA), manifest and cycloplegic refraction, slit-lamp biomicroscopy, dilated fundus examination, and intraocular pressure measurement. Mesopic contrast sensitivity was assessed using the CSV-1000, and anterior corneal aberrations were measured using the Sirius CSO + tomographer (CSO, Italy).

Postoperative follow-up examinations were scheduled for 1 week, 1 month, and 3 months. At each visit, UDVA, CDVA, manifest refraction, mesopic contrast sensitivity, slit-lamp examination findings, and corneal tomography were recorded.

### Surgical technique

The SMILE PRO surgery using the VISUMAX 800 femtosecond laser system (Carl Zeiss Meditec, Jena, Germany) was set up with a cap thickness of 100 μm, a cap diameter of 7.5–7.8 mm, and an optical zone of 6.0–6.8 mm, depending on individual refractive parameters. Laser settings included a pulse energy of 140 nJ, spot spacing of 4.4 μm, and track spacing of 3.8 μm for both cap and lenticule creation. No manual preoperative corneal marking was performed.

A 2.0-mm incision was created at the 120-degree position, with an incision angle of 29 degrees and a side-cut angle of 90 degrees. The lenticule was dissected using a Chansue dissector and extracted through the small corneal incision according to standard SMILE technique.

### Intraoperative optical zone, decentration, and cyclotorsion assessment

The intraoperatively selected optical zone diameter was recorded from the VISUMAX 800 surgical records.

Intraoperative centration was assessed using the CentraLign system integrated into the VISUMAX 800 platform. For the purpose of this study, centration was operationalized as the radial decentration magnitude, defined as the displacement between the intended treatment center and the achieved lenticule center in the corneal treatment plane. The intended treatment center was determined after CentraLign alignment and corresponded clinically to the surgeon-selected visual-axis reference during docking.

Geometrically, radial decentration can be expressed as d= $\sqrt{x^{2}+y^{2}}$ , where x and y represent horizontal and vertical displacement, respectively. In the exported surgical records used for this study, only radial decentration magnitude was available; directional x-y components were not exported and therefore were not analyzed.

Recorded decentration values were exported in 0.1-mm increments and were categorized as 0.0 mm, 0.1 mm, and ≥ 0.2 mm for the primary analysis. These categories reflected the rounded values available from the surgical records rather than biologically distinct thresholds.

Cyclotorsion was assessed using the OcuLign iris-registration system and was defined as the rotational difference between the preoperative reference image and the intraoperative eye position during docking. Cyclotorsion was recorded in degrees, and absolute values were used for analysis to reflect the magnitude of rotational misalignment regardless of direction.

### Outcome measures

The primary outcomes of this study were visual quality parameters, including mesopic contrast sensitivity and anterior corneal wavefront aberrations.

Mesopic contrast sensitivity was measured using the CSV-1000 system at spatial frequencies of 1.5, 3, 6, 12, and 18 cycles per degree. Before testing, patients underwent 10–15 min of adaptation in a dim room with ambient illumination of approximately 15 lux. Testing was performed at a distance of 2.5 m, with target luminance maintained at 3 cd/m^2^, to simulate low-light visual conditions relevant to night-time visual tasks. Anterior corneal wavefront aberrations were obtained using the Sirius CSO + tomographer and calculated for a standardized 6-mm pupil diameter using the device software. Measurements with poor acquisition quality were repeated according to the device quality-control criteria. Total ocular wavefront aberrometry was not included in the standardized clinical protocol and was therefore not available for analysis. Accordingly, the optical outcomes in this study represent anterior corneal wavefront changes rather than total ocular aberrations or internal optical aberrations.

Visual acuity (UDVA and CDVA) was included as a secondary outcome to provide a clinical context for visual performance. The safety index was calculated as postoperative CDVA divided by preoperative CDVA, and the efficacy index was calculated as postoperative UDVA divided by preoperative CDVA, using decimal visual acuity values.

Intraoperative centration and cyclotorsion parameters were recorded from the VISUMAX 800 surgical records and analyzed as potential factors associated with postoperative optical quality.

In the present analysis, visual acuity and manifest refraction were used only as supportive clinical context. The primary outcomes were contrast sensitivity and anterior corneal aberration parameters, whereas refractive predictability and astigmatic vector outcomes were addressed separately in the companion manuscript.

### Statistical analysis

Statistical analyses were performed using SPSS software (version 20.0; IBM Corp., Armonk, NY, USA). Continuous variables were reported as mean ± standard deviation, and categorical variables as frequencies and percentages.

Because both eyes from the same patient could be included, generalized estimating equations (GEE) were used to account for inter-eye correlation. Patient identification number was specified as the subject variable, eye laterality as the within-subject variable, and an exchangeable working correlation structure was selected because each patient could contribute a maximum of two eyes and within-patient inter-eye correlation was expected between paired eyes. Robust standard errors were used to reduce the influence of possible misspecification of the working correlation structure.

Multivariable GEE models were constructed for 3-month postoperative anterior corneal aberration outcomes and contrast sensitivity outcomes. Candidate predictors were prespecified based on clinical relevance and included age, sex, preoperative spherical equivalent, preoperative cylinder, astigmatism severity group, intraoperatively selected optical zone diameter, mesopic pupil size, intraoperative decentration category, and absolute cyclotorsion. In an additional sensitivity analysis, recorded decentration magnitude was also analyzed as a continuous predictor in 0.1-mm increments instead of decentration category.

For contrast sensitivity, five frequency-specific GEE models were fitted, corresponding to approximately 45 covariate-frequency associations across the tested spatial frequencies and prespecified covariates. No formal adjustment for multiplicity was applied. Therefore, nominal two-sided *p* values <0.05 are reported for descriptive and exploratory purposes only and should not be interpreted as confirmatory evidence. These frequency-specific analyses were considered exploratory and hypothesis-generating, and the identified associations require validation in independent cohorts before clinical interpretation. For categorical predictors, overall Type III Wald tests were used for inference, while pairwise coefficients were reported in the Supplementary Tables according to the specified reference categories. In the main tables, predictors with nominal *p* < 0.05 are presented; for categorical predictors, *p* values represent overall Type III Wald tests. Complete GEE outputs, including nonsignificant covariates, standard errors, confidence intervals, Wald *χ*^2^ statistics, and *p* values, are provided as Supplementary material.

## Results

### Patient demographics and surgical characteristics

A total of 160 eyes from 102 patients were included (mean age: 23.7 ± 4.7 years, 63.7% female). Baseline characteristics are summarized in Table 1. Preoperative astigmatism severity was divided into groups in Table 2. The distribution of astigmatism severity was uneven, with most eyes falling within the lower range of high astigmatism. Therefore, subgroup comparisons should be interpreted as exploratory and not as evidence of equivalence.

**Table 1:** Baseline and intraoperative characteristics.

Baseline characteristics	Values, mean ± SD (range)
Sphere (D)	−6.00 ± 2.00 (−0.50 to −10.00)
Cylinder (D)	2.51 ± 0.56 (2.00 to 5.00)
Spherical equivalent (D)	−7.21 ± 2.03 (−2.13 to −12.00)
UDVA (LogMAR)	1.4 ± 0.16 (0.4 to 1.7)
CDVA (LogMAR)	0.02 ± 0.03 (0.0 to 0.1)
IOP (mmHg)	16.1 ± 2.75 (10 to 20)
Mesopic pupil size (mm)	6.23 ± 0.78 (3.43 to 8.23)
Total HOAs (μm, 6-mm pupil)	0.37 ± 0.13
SA (μm, 6-mm pupil)	0.17 ± 0.06
Coma (μm, 6-mm pupil)	0.21 ± 0.11

Intraoperative characteristics	Values
Right eye, n (%)	72 (45.0)
Left eye, n (%)	88 (55.0)
Intraoperative optical zone diameter (mm)	6.63 ± 0.27 (6.0 to 6.8)
Recorded decentration magnitude (mm)	0.14 ± 0.09 (0.00 to 0.50)
Recorded decentration 0.0 mm, n (%)	28 (17.5)
Recorded decentration 0.1 mm, n (%)	121 (75.6)
Recorded decentration ≥ 0.2 mm, n (%)	11 (6.9)

SD, standard deviation; D, diopters; UDVA, uncorrected distance visual acuity; CDVA, corrected distance visual acuity; IOP, intraocular pressure; HOAs, Higher-Order Aberrations; SA, Spherical Aberration.

**Table 2:** Preoperative astigmatism severity.

Group	Preoperative magnitude (D)	Percentage (eyes)
Group 1	2.00 to 2.75 D	85% (136/160)
Group 2	3.00 to 3.75 D	10.6% (17/160)
Group 3	4.00 to 5.00 D	4.4% (7/160)

D, diopters.

Among the 102 patients, 58 patients (56.9%) contributed both eyes and 44 patients (43.1%) contributed one eye. The eye-level dataset included 72 right eyes (45.0%) and 88 left eyes (55.0%). The intraoperatively selected optical zone diameter was 6.63 ± 0.27 mm, ranging from 6.0 to 6.8 mm. The mean recorded decentration magnitude was 0.14 ± 0.09 mm, ranging from 0.00 to 0.50 mm. Based on values exported from the VISUMAX 800 surgical record, decentration was recorded as 0.0 mm in 28 eyes (17.5%), 0.1 mm in 121 eyes (75.6%), and ≥ 0.2 mm in 11 eyes (6.9%).

### Visual and refractive outcomes

At 3 months, refractive outcomes demonstrated high accuracy and stability. The mean postoperative spherical equivalent was close to emmetropia, and 98.8% of eyes achieved a UDVA of 20/25 or better.

Safety and efficacy indices (1.01 and 0.99, respectively) supported favorable short-term visual outcomes in this cohort.

Residual astigmatism was low (−0.52 ± 0.28 D) and did not differ significantly among preoperative astigmatism subgroups (Wald *χ*^2^ = 0.665, df = 2, *p* = 0.717), indicating consistent astigmatic correction across different levels of cylindrical error. The distribution of residual refractive astigmatism is presented in Supplementary Figure S1, with 69% of eyes within ≤ 0.50 D and 98% within ≤ 1.00 D at 3 months.

### Changes in anterior corneal wavefront aberrations

**Figure 1:**
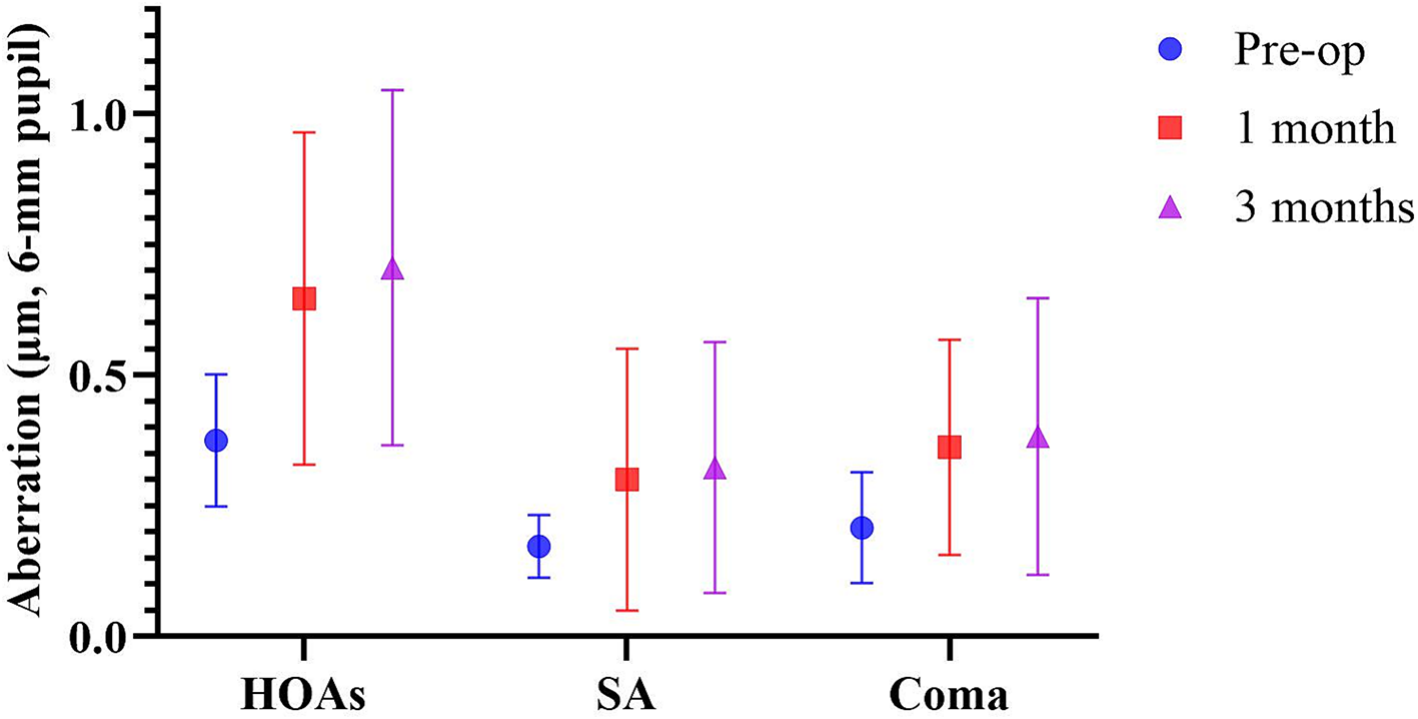
Changes in anterior corneal aberrations, including total higher-order aberrations, spherical aberration, and coma, from preoperative to 1 month and 3 months postoperatively under a 6-mm pupil. Error bars represent standard deviation and are intended to show descriptive variability. Preoperative and 3-month data were available for 160 eyes, and 1-month data were available for 96 eyes because of incomplete attendance at the intermediate follow-up visit. HOAs, higher-order aberrations; SA, spherical aberration.

At 1 month, total HOAs (0.65 ± 0.32 μm), coma (0.36 ± 0.21 μm), and spherical aberration (0.29 ± 0.25 μm), all measured at a 6-mm pupil, increased compared with preoperative values and remained elevated through 3 months (Figure 1). One-month aberration data were available for 96 eyes because of incomplete attendance at the intermediate follow-up visit. Although these changes were consistent with postoperative induction of anterior corneal aberrations, their clinical significance should be interpreted cautiously because patient-reported quality-of-vision outcomes were not assessed.

Key multivariable associations with 3-month anterior corneal aberration outcomes are summarized in Table 3, and the complete model outputs are provided in Supplementary Table S1. Preoperative astigmatism subgroup was not significantly associated with postoperative total HOAs, coma, or spherical aberration in the multivariable models. A larger intraoperatively selected optical zone diameter was consistently associated with lower values of total HOAs, coma, and spherical aberration. Preoperative spherical equivalent was associated with total HOAs and spherical aberration, whereas absolute cyclotorsion was associated only with coma. Decentration category was not significantly associated with anterior corneal aberration outcomes.

**Table 3:** Key multivariable associations with postoperative anterior corneal aberrations at 3 months.

Outcome	Predictor	*β*	95% CI	*p* value
Total HOAs, 6-mm pupil	Preoperative spherical equivalent	−0.034	−0.060 to −0.009	0.009
Total HOAs, 6-mm pupil	Intraoperative optical zone diameter	−0.491	−0.724 to −0.259	<0.001
Coma, 6-mm pupil	Absolute cyclotorsion	0.020	0.001 to 0.040	0.036
Coma, 6-mm pupil	Intraoperative optical zone diameter	−0.301	−0.495 to −0.107	0.002
Spherical aberration, 6-mm pupil	Preoperative spherical equivalent	−0.023	−0.040 to −0.007	0.006
Spherical aberration, 6-mm pupil	Intraoperative optical zone diameter	−0.346	−0.513 to −0.178	<0.001

Only nominal associations with *p* < 0.05 are shown. For categorical predictors, P values represent overall Type III Wald tests. Full model outputs, including nonsignificant covariates, are provided in Supplementary Table S1. GEE, generalized estimating equations; HOAs, higher-order aberrations; CI, confidence interval.

### Changes in contrast sensitivity

**Figure 2:**
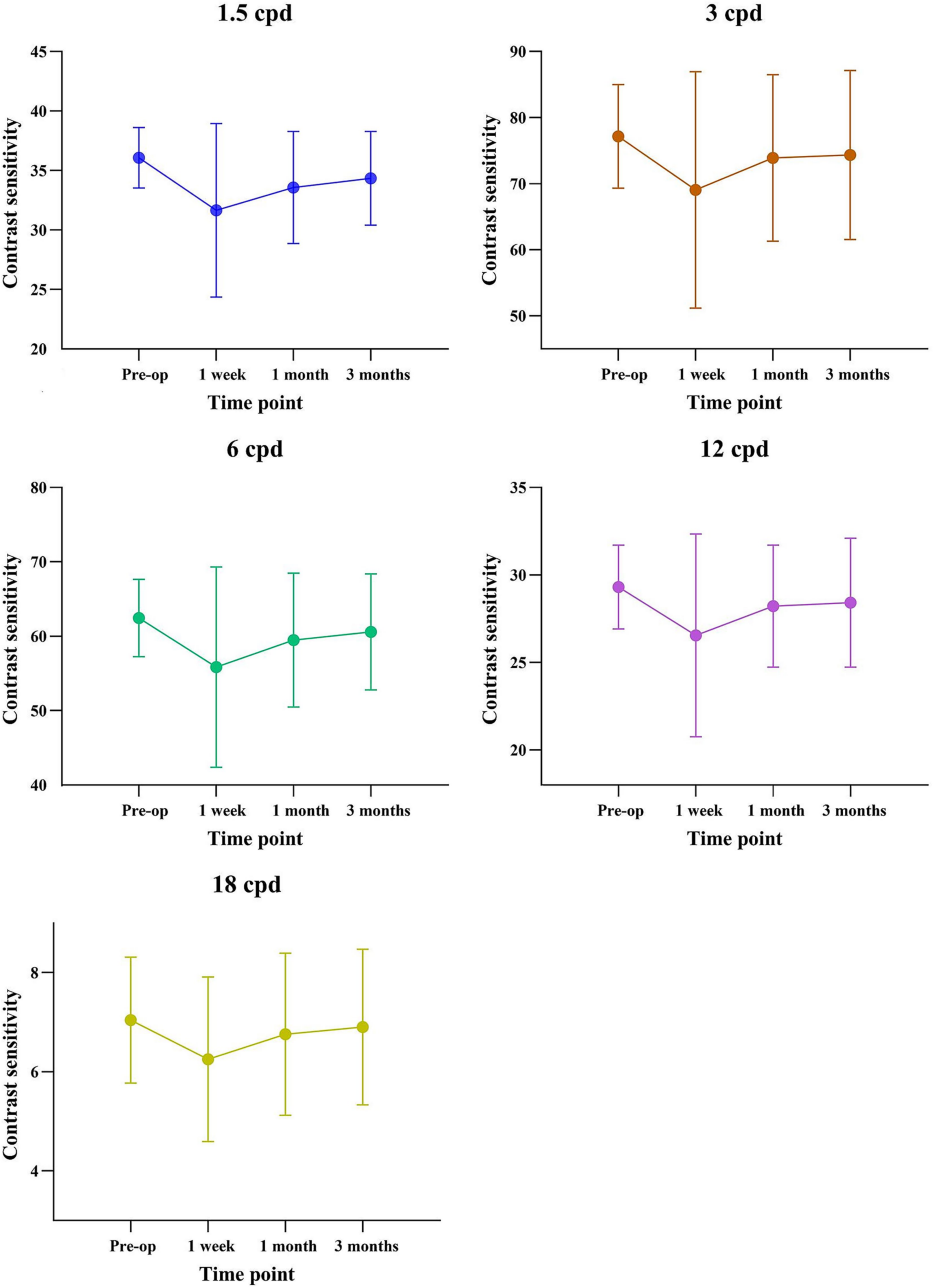
Mesopic contrast sensitivity at baseline and postoperative time points across spatial frequencies from 1.5 to 18 cycles per degree. Error bars represent standard deviation and are intended to show descriptive variability. Data were available for 160 eyes preoperatively, 143 eyes at 1 week, 103 eyes at 1 month, and 160 eyes at 3 months. Y-axis ranges differ across panels to improve visualization of temporal changes within each spatial frequency. Cpd = cycles per degree.

Figure 2 shows the postoperative changes in mesopic contrast sensitivity across spatial frequencies. Key multivariable associations with 3-month postoperative contrast sensitivity are summarized in Table 4, and the complete model outputs are provided in Supplementary Table S2. For contrast sensitivity outcomes, *β* coefficients represent the expected change in CSV-1000 contrast sensitivity score per 1.00 D increase in the corresponding refractive predictor, holding other covariates constant. Because these values are expressed in instrument-specific score units and patient-reported visual quality was not assessed, their direct clinical significance should be interpreted cautiously. The absolute differences across spatial frequencies were modest; therefore, these findings should be interpreted as exploratory rather than definitive evidence of clinically meaningful frequency-specific effects.

**Table 4:** Key multivariable associations with postoperative contrast sensitivity at 3 months.

Spatial frequency	Predictor	*β*	95% CI	*p* value
3 cpd	Preoperative spherical equivalent	1.720	0.412 to 3.027	0.010
3 cpd	Preoperative cylinder	8.252	0.893 to 15.611	0.028
6 cpd	Preoperative spherical equivalent	1.034	0.235 to 1.832	0.011
6 cpd	Preoperative cylinder	4.289	0.127 to 8.451	0.043
12 cpd	Decentration category	–	–	0.034*
18 cpd	Preoperative spherical equivalent	0.159	0.022 to 0.296	0.023

Only nominal associations with *p* < 0.05 are shown. For categorical predictors, P values represent overall Type III Wald tests, and the reference category was recorded decentration ≥0.2 mm. Pairwise coefficients depend on the selected reference category. Full model outputs are provided in Supplementary Table S2. GEE, generalized estimating equations; cpd, cycles per degree; CI, confidence interval. *p value from the overall Type III Wald test for the categorical predictor.

Preoperative astigmatism subgroup was not significantly associated with contrast sensitivity at any tested spatial frequency. At 1.5 cpd, no covariate showed a robust association with postoperative contrast sensitivity. At intermediate spatial frequencies, preoperative spherical equivalent and cylinder showed nominal associations with contrast sensitivity at both 3 cpd and 6 cpd. At 12 cpd, decentration category showed an overall nominal association with contrast sensitivity (*p* = 0.034); however, pairwise coefficients depended on the selected reference category. Because decentration was exported in 0.1-mm increments, a sensitivity analysis was performed using recorded decentration magnitude as a continuous predictor instead of decentration category. In this analysis, recorded decentration magnitude was not significantly associated with 12-cpd contrast sensitivity at 3 months (*β* = 2.901, 95% CI: −2.791 to 8.592; *p* = 0.318). At 18 cpd, preoperative spherical equivalent was the only covariate showing a nominal association. Given the exploratory nature of the spatial-frequency analyses and the absence of adjustment for multiple comparisons, these associations should be interpreted cautiously.

In addition, mesopic pupil size was included as a clinically relevant covariate in the multivariable GEE models but was not significantly associated with postoperative anterior corneal aberration or contrast sensitivity outcomes. Detailed coefficients for mesopic pupil size and other nonsignificant covariates are provided in Supplementary Tables S1, S2.

## Discussion

### Principal findings

This study suggests that visual quality outcomes following SMILE PRO in eyes with high astigmatism may reflect distinct associations across outcome measures and spatial frequencies.

Three principal findings emerged. First, within the astigmatism range and subgroup distribution represented in this cohort, no statistically significant differences in visual quality outcomes were observed among preoperative astigmatism subgroups. Second, wavefront aberrations were mainly associated with optical zone diameter and spherical refractive magnitude, with coma showing an additional association with cyclotorsion. Third, contrast sensitivity showed exploratory frequency-specific associations, suggesting that different optical and refractive factors may contribute differently across spatial frequencies.

### Associations with astigmatism magnitude

Despite traditional concerns regarding the correction of high astigmatism (8), within the distribution of astigmatism severity represented in this cohort, no statistically significant differences in visual quality outcomes were observed among astigmatism subgroups. Because relatively few eyes were included in the highest astigmatism subgroup, this finding should not be interpreted as definitive evidence that astigmatism magnitude does not affect visual quality.

Within the treated range, these findings suggest that categorical astigmatism severity was not a dominant explanatory factor for short-term visual quality outcomes in this cohort. However, this interpretation should be cautious because the subgroup distribution was imbalanced. Improved lenticule extraction precision and integrated cyclotorsion compensation may partly reduce axis-related error, but the present study was not designed to directly test this mechanism.

Importantly, continuous refractive variables, such as spherical equivalent and cylinder, were associated with contrast sensitivity at intermediate spatial frequencies, indicating that refractive effects follow a continuous rather than threshold-dependent pattern.

### Associations with wavefront aberrations

Higher-order aberrations (HOAs) tend to increase after laser refractive surgery, whereas coma and spherical aberration have greater impact on the optical quality than other HOAs (11). Several mechanisms may explain the induction of HOAs, including irregular astigmatism, a more oblate corneal shape, decentration, and a smaller optical zone, as well as larger pupil size (12).

Previous studies have suggested that SMILE may induce fewer higher-order aberrations than LASIK or PRK under low-light conditions, which may contribute to better subjective visual tolerance in some patients (13). However, such comparisons should be interpreted as indirect contextual evidence, because the present study did not include an internal comparator group such as conventional SMILE, LASIK, PRK, or eyes with lower astigmatism. In the present cohort, an expected increase in anterior corneal aberrations was observed after SMILE PRO. Whether the magnitude of these changes is clinically meaningful remains uncertain, as this study did not include validated subjective quality-of-vision questionnaires or total ocular aberration measurements. Eyes with higher degrees of myopia exhibited greater postoperative aberrations, consistent with the increased stromal alteration and smaller optical zone diameter required for higher refractive correction. Our findings are comparable to those reported by Zhou et al.; however, those authors also noted that in the long term, the 6.5 mm optical zone had no effect on the subjective optical quality of the patients (14).

Displacement between the corneal apex and visual axis has been associated with higher postoperative HOAs after SMILE (15), emphasizing the potential importance of treatment centration. This is also consistent with Hou et al. (5), who reported that SMILE may induce more vertical coma in association with vertical decentration; however, SMILE surgery lacked automated guidance from CentraLign and OcuLign systems. This study observed that cyclotorsion was specifically associated with coma, supporting the concept that rotational misalignment may induce asymmetric aberrations. In contrast, centration was not significantly associated with global aberration measures, suggesting that these metrics may be relatively less sensitive to minor decentration in this setting.

### Frequency-dependent behavior of contrast sensitivity

Contrast sensitivity may fluctuate during the early postoperative periods after SMILE. It may decrease early after surgery and return toward preoperative levels within the first month, which is compatible with early functional recovery after SMILE (16). Tian et al. indicated that contrast sensitivity remained consistent under varying lighting conditions after 5 years of SMILE surgery (17). Chansue et al. (18) suggested potential improvements in contrast sensitivity following SMILE under long-term observation.

Postoperative contrast sensitivity also showed a frequency-specific pattern. In this cohort, most changes occurred during the early postoperative period, with apparent stabilization after 1 month, consistent with previous reports of functional recovery after SMILE (16, 17).

At 1.5 cpd, contrast sensitivity was largely unaffected by optical or refractive variables. This may indicate that low-frequency contrast sensitivity is less vulnerable to the level of optical irregularity observed in this cohort (19).

At intermediate spatial frequencies (3–6 cpd), contrast sensitivity showed nominal associations with refractive magnitude, suggesting a possible contribution of refractive correction to functional visual performance, consistent with previous reports (20, 21). This finding may be interpreted in the context of prior work suggesting that preoperative refractive status can influence postoperative contrast sensitivity after SMILE (22). However, because the present analysis evaluated absolute 3-month contrast sensitivity rather than change from baseline, this association should not be interpreted as evidence of greater postoperative improvement in eyes with higher preoperative refractive error. In addition, because contrast sensitivity reflects the combined influence of optical, refractive, neural, tear-film, and measurement-related factors, the observed effect sizes should be interpreted as modest exploratory associations rather than isolated optical mechanisms.

The association between decentration category and contrast sensitivity at 12 cpd may reflect the sensitivity of higher spatial frequencies to subtle optical misalignment. Even small degrees of decentration may alter the effective optical zone and induce asymmetric aberrations, particularly at higher spatial frequencies. Similar frequency-dependent vulnerability has been reported by Cao et al. (23), who observed reduced scotopic contrast sensitivity at 3, 6, and 12 cpd despite stable photopic conditions, suggesting a greater impact of higher-order aberrations under larger pupil conditions. However, in the present study, this association was not retained in the sensitivity analysis using recorded decentration magnitude as a continuous predictor; therefore, the decentration-related finding should be considered exploratory.

At 18 cpd, decentration category was not significantly associated with contrast sensitivity. This pattern may suggest that factors beyond treatment alignment contribute more prominently at the highest spatial frequency, which is compatible with previous evidence (19, 24).

Together, these findings suggest a possible frequency-specific pattern in the associations with contrast sensitivity. However, because this was an observational study without experimental manipulation of optical or neural factors, the proposed interpretation should be considered exploratory rather than mechanistic. Given the relatively large number of covariate-frequency combinations evaluated, some nominal associations may have arisen by chance. Therefore, these findings should be regarded as hypothesis-generating and require confirmation in independent cohorts.

Alternative explanations should also be considered. Because contrast sensitivity depends on optical, refractive, and non-optical factors, its relationship with anterior corneal aberrations should not be assumed to be direct or one-to-one. Residual refractive status, pupil size, tear-film stability, retinal and neural processing, and test–retest variability may all contribute to the observed outcomes. This may partly explain why contrast sensitivity outcomes were not fully mirrored by anterior corneal aberration metrics in the present study. In particular, small numerical differences at higher spatial frequencies under mesopic conditions should be interpreted cautiously and should not necessarily be regarded as clinically meaningful changes.

### Clinical implications

These findings are compatible with generally stable short-term visual quality outcomes after SMILE PRO in eyes with high astigmatism within the range studied. The consistent association between optical zone diameter and anterior corneal aberrations supports careful optical-zone planning when sufficient corneal tissue is available.

The observed associations involving cyclotorsion and decentration category suggest that intraoperative alignment may remain relevant for specific optical and functional outcomes, particularly coma and fine spatial visual performance. However, these observational findings do not establish that additional intraoperative adjustment beyond the alignment protocol used in the present study would improve visual quality, and larger comparative studies with longer follow-up are needed before specific clinical recommendations can be made.

### Limitations

This study has several limitations. First, it was conducted at a single center with a single surgeon, which may limit the generalizability of the findings. In addition, although high astigmatism was predefined as manifest cylinder ≥ 2.00 D, relatively few eyes were included in the highest cylinder range. This imbalance may have reduced the power to detect subgroup-specific effects among eyes with very high astigmatism. Second, the follow-up period was limited to 3 months. Although refractive and corneal optical parameters often stabilize within the early postoperative period after SMILE, contrast sensitivity and patient-perceived visual quality may continue to evolve over longer follow-up. Therefore, the present findings should be interpreted as short-term outcomes.

Third, only anterior corneal aberrations were evaluated; therefore, the optical analysis does not capture internal ocular aberrations or total ocular wavefront error. Because contrast sensitivity reflects both optical and neural processing, the observed functional visual performance cannot be attributed solely to anterior corneal optics. Thus, the aberration results should be interpreted as anterior corneal optical changes rather than total ocular optical quality. Patient-reported quality-of-vision questionnaires were not included; therefore, the clinical relevance of the observed aberration changes could not be directly linked to subjective visual symptoms. However, the cornea is known to account for the majority of ocular aberrations (25), and SMILE PRO primarily alters anterior corneal structure, supporting the relevance of these measurements.

In addition, visual acuity was assessed using a Snellen chart rather than a logMAR chart, which is less optimal for research-level acuity measurement. However, visual acuity was included only as a secondary clinical outcome, and all measurements were obtained under standardized conditions by the same examiner.

Furthermore, decentration was analyzed using recorded radial magnitude in 0.1-mm increments, and directional x-y components were not available in the exported clinical dataset. This may have reduced sensitivity for detecting direction-specific effects of treatment displacement on coma and contrast sensitivity.

Finally, no comparator group was included; therefore, direct comparisons with conventional SMILE or excimer laser-based procedures could not be made.

## Conclusion

At 3 months after SMILE PRO in eyes with high astigmatism, postoperative anterior corneal aberration and contrast sensitivity outcomes showed exploratory associations with refractive magnitude and intraoperatively selected optical zone diameter, with additional findings related to cyclotorsion and decentration category. However, the absence of significant subgroup associations should be interpreted cautiously because the astigmatism subgroups were imbalanced and the number of eyes with very high astigmatism was limited. Anterior corneal aberrations were mainly associated with optical zone diameter and spherical refractive error, whereas coma showed an additional association with cyclotorsion. Contrast sensitivity demonstrated exploratory frequency-dependent associations, with refractive factors contributing at intermediate spatial frequencies and decentration category showing a modest overall association at 12 cpd. These short-term findings should be interpreted cautiously and require confirmation in larger comparative studies with longer follow-up.

## Data Availability

The raw data supporting the conclusions of this article will be made available by the authors, without undue reservation.
