# Supplementary material for: Frequency-dependent associations of contrast sensitivity and corneal aberrations after SMILE PRO in eyes with high astigmatism
Source: Front Med (Lausanne). 2026 Jul 20;13:1867550. doi: 10.3389/fmed.2026.1867550 (PMC13430464; doi:10.3389/fmed.2026.1867550)
Supplement: Supplementary file 1 [file Table_1.DOCX]

Supplementary Figure S1. Distribution of residual refractive astigmatism at 3 months postoperatively.

Abbreviations: D = diopter.

Supplementary Table S1. Full multivariable GEE output for postoperative anterior corneal aberrations at 3 months.

| **Outcome** | **Predictor** | **β** | **SE** | **95% CI** | **Wald χ²** | **P value** |
| --- | --- | --- | --- | --- | --- | --- |
| **HOAs 6 mm** | | | | | | |
| HOAs 6 mm | centra_ord = 1 | -0.042 | 0.0929 | -0.224 to 0.140 | 0.204 | 0.651 |
| HOAs 6 mm | centra_ord = 2 | -0.001 | 0.0984 | -0.194 to 0.191 | 0.000 | 0.989 |
| HOAs 6 mm | centra_ord = 3 | Reference | - | - | - | - |
| HOAs 6 mm | Sex = 0 | -0.064 | 0.0547 | -0.171 to 0.044 | 1.351 | 0.245 |
| HOAs 6 mm | Sex = 1 | Reference | - | - | - | - |
| HOAs 6 mm | GroupCYL_pre = 1 | 0.003 | 0.2155 | -0.420 to 0.425 | 0.000 | 0.989 |
| HOAs 6 mm | GroupCYL_pre = 2 | -0.148 | 0.1921 | -0.525 to 0.228 | 0.596 | 0.440 |
| HOAs 6 mm | GroupCYL_pre = 3 | Reference | - | - | - | - |
| HOAs 6 mm | Age | -0.002 | 0.0054 | -0.013 to 0.008 | 0.208 | 0.648 |
| HOAs 6 mm | Cyclo_abs | 0.004 | 0.0116 | -0.019 to 0.027 | 0.124 | 0.725 |
| HOAs 6 mm | SEQ_pre | -0.034 | 0.0130 | -0.060 to -0.009 | 6.881 | 0.009 |
| HOAs 6 mm | CYL_pre | -0.103 | 0.0694 | -0.239 to 0.033 | 2.219 | 0.136 |
| HOAs 6 mm | PPs_in | -0.017 | 0.0352 | -0.086 to 0.052 | 0.226 | 0.635 |
| HOAs 6 mm | OZ_in | -0.491 | 0.1187 | -0.724 to -0.259 | 17.141 | <0.001 |
| **Coma 6 mm** | | | | | | |
| Coma 6 mm | centra_ord = 1 | 0.007 | 0.0871 | -0.164 to 0.177 | 0.006 | 0.938 |
| Coma 6 mm | centra_ord = 2 | 0.078 | 0.0937 | -0.106 to 0.261 | 0.688 | 0.407 |
| Coma 6 mm | centra_ord = 3 | Reference | - | - | - | - |
| Coma 6 mm | Sex = 0 | 0.007 | 0.0413 | -0.074 to 0.088 | 0.030 | 0.863 |
| Coma 6 mm | Sex = 1 | Reference | - | - | - | - |
| Coma 6 mm | GroupCYL_pre = 1 | -0.106 | 0.2096 | -0.517 to 0.305 | 0.257 | 0.612 |
| Coma 6 mm | GroupCYL_pre = 2 | -0.234 | 0.1906 | -0.607 to 0.140 | 1.505 | 0.220 |
| Coma 6 mm | GroupCYL_pre = 3 | Reference | - | - | - | - |
| Coma 6 mm | Age | -0.001 | 0.0044 | -0.010 to 0.008 | 0.045 | 0.833 |
| Coma 6 mm | Cyclo_abs | 0.020 | 0.0098 | 0.001 to 0.040 | 4.407 | 0.036 |
| Coma 6 mm | SEQ_pre | -0.006 | 0.0113 | -0.028 to 0.017 | 0.238 | 0.626 |
| Coma 6 mm | CYL_pre | -0.025 | 0.0659 | -0.154 to 0.104 | 0.144 | 0.704 |
| Coma 6 mm | PPs_in | 0.030 | 0.0314 | -0.031 to 0.091 | 0.916 | 0.339 |
| Coma 6 mm | OZ_in | -0.301 | 0.0988 | -0.495 to -0.107 | 9.274 | 0.002 |
| **Spherical aberration 6 mm** | | | | | | |
| Spherical aberration 6 mm | centra_ord = 1 | 0.021 | 0.0353 | -0.048 to 0.090 | 0.349 | 0.555 |
| Spherical aberration 6 mm | centra_ord = 2 | 0.004 | 0.0415 | -0.077 to 0.086 | 0.011 | 0.917 |
| Spherical aberration 6 mm | centra_ord = 3 | Reference | - | - | - | - |
| Spherical aberration 6 mm | Sex = 0 | -0.046 | 0.0395 | -0.124 to 0.031 | 1.378 | 0.240 |
| Spherical aberration 6 mm | Sex = 1 | Reference | - | - | - | - |
| Spherical aberration 6 mm | GroupCYL_pre = 1 | 0.066 | 0.0878 | -0.106 to 0.238 | 0.572 | 0.450 |
| Spherical aberration 6 mm | GroupCYL_pre = 2 | 0.118 | 0.0734 | -0.026 to 0.262 | 2.586 | 0.108 |
| Spherical aberration 6 mm | GroupCYL_pre = 3 | Reference | - | - | - | - |
| Spherical aberration 6 mm | Age | -0.003 | 0.0035 | -0.010 to 0.004 | 0.613 | 0.434 |
| Spherical aberration 6 mm | Cyclo_abs | -0.006 | 0.0078 | -0.021 to 0.009 | 0.591 | 0.442 |
| Spherical aberration 6 mm | SEQ_pre | -0.023 | 0.0086 | -0.040 to -0.007 | 7.455 | 0.006 |
| Spherical aberration 6 mm | CYL_pre | -0.001 | 0.0383 | -0.076 to 0.074 | 0.000 | 0.988 |
| Spherical aberration 6 mm | PPs_in | 0.025 | 0.0218 | -0.018 to 0.068 | 1.311 | 0.252 |
| Spherical aberration 6 mm | OZ_in | -0.346 | 0.0855 | -0.513 to -0.178 | 16.327 | <0.001 |

Abbreviations: GEE = generalized estimating equations; β = regression coefficient; SE = robust standard error; CI = confidence interval; Wald χ² = Wald chi-square statistic; HOAs = higher-order aberrations; SEQ = spherical equivalent; CYL = cylinder; PPs = mesopic pupil size; OZ = intraoperatively selected optical zone diameter.

Variable definitions: SEQ_pre = preoperative spherical equivalent; CYL_pre = preoperative cylinder; PPs_in = mesopic pupil size; OZ_in = intraoperatively selected optical zone diameter; Cyclo_abs = absolute cyclotorsion; centra_ord = decentration category; GroupCYL_pre = preoperative astigmatism severity group.

GroupCYL_pre was categorized as follows: group 1, 2.00 to 2.75 D; group 2, 3.00 to 3.75 D; group 3, 4.00 to 5.00 D. centra_ord was categorized as follows: 1, <0.1 mm; 2, 0.1 mm; 3, ≥0.2 mm. Reference categories were GroupCYL_pre = 3, centra_ord = 3, and Sex = 1.

Supplementary Table S2. Full multivariable GEE output for postoperative contrast sensitivity at 3 months.

| **Spatial frequency** | **Predictor** | **β** | **SE** | **95% CI** | **Wald χ²** | **P value** |
| --- | --- | --- | --- | --- | --- | --- |
| **1.5 cpd** | | | | | | |
| 1.5 cpd | centra_ord = 1 | -2.054 | 1.2990 | -4.601 to 0.492 | 2.501 | 0.114 |
| 1.5 cpd | centra_ord = 2 | -2.440 | 1.1194 | -4.634 to -0.246 | 4.752 | 0.029 |
| 1.5 cpd | centra_ord = 3 | Reference | - | - | - | - |
| 1.5 cpd | Sex = 0 | 0.179 | 0.7537 | -1.299 to 1.656 | 0.056 | 0.813 |
| 1.5 cpd | Sex = 1 | Reference | - | - | - | - |
| 1.5 cpd | GroupCYL_pre = 1 | 4.134 | 2.6896 | -1.137 to 9.406 | 2.363 | 0.124 |
| 1.5 cpd | GroupCYL_pre = 2 | 4.730 | 2.4594 | -0.090 to 9.550 | 3.699 | 0.054 |
| 1.5 cpd | GroupCYL_pre = 3 | Reference | - | - | - | - |
| 1.5 cpd | Age | 0.000 | 0.0874 | -0.171 to 0.171 | 0.000 | 0.999 |
| 1.5 cpd | Cyclo_abs | -0.114 | 0.1496 | -0.407 to 0.179 | 0.579 | 0.447 |
| 1.5 cpd | SEQ_pre | 0.333 | 0.1832 | -0.026 to 0.692 | 3.314 | 0.069 |
| 1.5 cpd | CYL_pre | 0.368 | 0.9274 | -1.450 to 2.185 | 0.157 | 0.692 |
| 1.5 cpd | PPs_in | -0.014 | 0.3913 | -0.781 to 0.753 | 0.001 | 0.972 |
| 1.5 cpd | OZ_in | -0.751 | 1.0708 | -2.849 to 1.348 | 0.492 | 0.483 |
| **3 cpd** | | | | | | |
| 3 cpd | centra_ord = 1 | -6.187 | 4.4297 | -14.869 to 2.495 | 1.951 | 0.162 |
| 3 cpd | centra_ord = 2 | -6.467 | 3.5856 | -13.494 to 0.561 | 3.253 | 0.071 |
| 3 cpd | centra_ord = 3 | Reference | - | - | - | - |
| 3 cpd | Sex = 0 | -0.776 | 2.1845 | -5.058 to 3.505 | 0.126 | 0.722 |
| 3 cpd | Sex = 1 | Reference | - | - | - | - |
| 3 cpd | GroupCYL_pre = 1 | -5.206 | 11.1300 | -27.020 to 16.608 | 0.219 | 0.640 |
| 3 cpd | GroupCYL_pre = 2 | 2.041 | 10.0848 | -17.724 to 21.807 | 0.041 | 0.840 |
| 3 cpd | GroupCYL_pre = 3 | Reference | - | - | - | - |
| 3 cpd | Age | 0.142 | 0.2208 | -0.291 to 0.574 | 0.412 | 0.521 |
| 3 cpd | Cyclo_abs | 0.123 | 0.4536 | -0.766 to 1.012 | 0.073 | 0.787 |
| 3 cpd | SEQ_pre | 1.720 | 0.6671 | 0.412 to 3.027 | 6.645 | 0.010 |
| 3 cpd | CYL_pre | 8.252 | 3.7546 | 0.893 to 15.611 | 4.830 | 0.028 |
| 3 cpd | PPs_in | 0.237 | 1.2531 | -2.219 to 2.693 | 0.036 | 0.850 |
| 3 cpd | OZ_in | -4.028 | 4.1814 | -12.223 to 4.168 | 0.928 | 0.335 |
| **6 cpd** | | | | | | |
| 6 cpd | centra_ord = 1 | -3.669 | 2.5927 | -8.750 to 1.413 | 2.002 | 0.157 |
| 6 cpd | centra_ord = 2 | -3.515 | 1.9453 | -7.327 to 0.298 | 3.264 | 0.071 |
| 6 cpd | centra_ord = 3 | Reference | - | - | - | - |
| 6 cpd | Sex = 0 | -0.104 | 1.3469 | -2.744 to 2.536 | 0.006 | 0.939 |
| 6 cpd | Sex = 1 | Reference | - | - | - | - |
| 6 cpd | GroupCYL_pre = 1 | -2.811 | 6.0989 | -14.765 to 9.142 | 0.212 | 0.645 |
| 6 cpd | GroupCYL_pre = 2 | -0.586 | 5.6892 | -11.737 to 10.565 | 0.011 | 0.918 |
| 6 cpd | GroupCYL_pre = 3 | Reference | - | - | - | - |
| 6 cpd | Age | -0.021 | 0.1477 | -0.310 to 0.269 | 0.020 | 0.888 |
| 6 cpd | Cyclo_abs | -0.071 | 0.3025 | -0.664 to 0.522 | 0.055 | 0.814 |
| 6 cpd | SEQ_pre | 1.034 | 0.4074 | 0.235 to 1.832 | 6.436 | 0.011 |
| 6 cpd | CYL_pre | 4.289 | 2.1235 | 0.127 to 8.451 | 4.080 | 0.043 |
| 6 cpd | PPs_in | -0.332 | 0.7884 | -1.877 to 1.213 | 0.177 | 0.674 |
| 6 cpd | OZ_in | -1.884 | 2.3853 | -6.559 to 2.791 | 0.624 | 0.430 |
| **12 cpd** | | | | | | |
| 12 cpd | centra_ord = 1 | -1.826 | 1.3002 | -4.374 to 0.723 | 1.972 | 0.160 |
| 12 cpd | centra_ord = 2 | -2.447 | 1.0379 | -4.481 to -0.413 | 5.558 | 0.018 |
| 12 cpd | centra_ord = 3 | Reference | - | - | - | - |
| 12 cpd | Sex = 0 | 1.110 | 0.8446 | -0.546 to 2.765 | 1.726 | 0.189 |
| 12 cpd | Sex = 1 | Reference | - | - | - | - |
| 12 cpd | GroupCYL_pre = 1 | 0.187 | 2.7282 | -5.160 to 5.534 | 0.005 | 0.945 |
| 12 cpd | GroupCYL_pre = 2 | 0.036 | 2.7507 | -5.355 to 5.427 | 0.000 | 0.990 |
| 12 cpd | GroupCYL_pre = 3 | Reference | - | - | - | - |
| 12 cpd | Age | -0.061 | 0.0714 | -0.201 to 0.079 | 0.734 | 0.391 |
| 12 cpd | Cyclo_abs | -0.026 | 0.1158 | -0.253 to 0.201 | 0.051 | 0.822 |
| 12 cpd | SEQ_pre | 0.299 | 0.1584 | -0.012 to 0.609 | 3.559 | 0.059 |
| 12 cpd | CYL_pre | 1.076 | 0.8902 | -0.669 to 2.821 | 1.461 | 0.227 |
| 12 cpd | PPs_in | -0.129 | 0.3394 | -0.794 to 0.536 | 0.145 | 0.704 |
| 12 cpd | OZ_in | -0.694 | 0.9775 | -2.610 to 1.222 | 0.504 | 0.478 |
| **18 cpd** | | | | | | |
| 18 cpd | centra_ord = 1 | -0.390 | 0.5026 | -1.375 to 0.596 | 0.601 | 0.438 |
| 18 cpd | centra_ord = 2 | -0.452 | 0.4375 | -1.309 to 0.406 | 1.066 | 0.302 |
| 18 cpd | centra_ord = 3 | Reference | - | - | - | - |
| 18 cpd | Sex = 0 | -0.167 | 0.2820 | -0.719 to 0.386 | 0.349 | 0.554 |
| 18 cpd | Sex = 1 | Reference | - | - | - | - |
| 18 cpd | GroupCYL_pre = 1 | 0.992 | 1.1270 | -1.216 to 3.201 | 0.775 | 0.379 |
| 18 cpd | GroupCYL_pre = 2 | 1.094 | 1.0774 | -1.018 to 3.206 | 1.031 | 0.310 |
| 18 cpd | GroupCYL_pre = 3 | Reference | - | - | - | - |
| 18 cpd | Age | 0.005 | 0.0325 | -0.059 to 0.069 | 0.025 | 0.873 |
| 18 cpd | Cyclo_abs | -0.019 | 0.0566 | -0.130 to 0.092 | 0.115 | 0.735 |
| 18 cpd | SEQ_pre | 0.159 | 0.0697 | 0.022 to 0.296 | 5.183 | 0.023 |
| 18 cpd | CYL_pre | 0.562 | 0.3680 | -0.160 to 1.283 | 2.330 | 0.127 |
| 18 cpd | PPs_in | -0.107 | 0.1708 | -0.442 to 0.228 | 0.393 | 0.531 |
| 18 cpd | OZ_in | -0.525 | 0.4364 | -1.380 to 0.330 | 1.448 | 0.229 |

Abbreviations: GEE = generalized estimating equations; β = regression coefficient; SE = robust standard error; CI = confidence interval; Wald χ² = Wald chi-square statistic; cpd = cycles per degree; SEQ = spherical equivalent; CYL = cylinder; PPs = mesopic pupil size; OZ = intraoperatively selected optical zone diameter.

Variable definitions: SEQ_pre = preoperative spherical equivalent; CYL_pre = preoperative cylinder; PPs_in = mesopic pupil size; OZ_in = intraoperatively selected optical zone diameter; Cyclo_abs = absolute cyclotorsion; centra_ord = decentration category; GroupCYL_pre = preoperative astigmatism severity group.

GroupCYL_pre was categorized as follows: group 1, 2.00 to 2.75 D; group 2, 3.00 to 3.75 D; group 3, 4.00 to 5.00 D. centra_ord was categorized as follows: 1, <0.1 mm; 2, 0.1 mm; 3, ≥0.2 mm. Reference categories were GroupCYL_pre = 3, centra_ord = 3, and Sex = 1.
